# Supplementary material for: The causal relationship between the human gut microbiota and pyogenic arthritis: a Mendelian randomization study
Source: Front Cell Infect Microbiol. 2024 Nov 26;14:1452480. doi: 10.3389/fcimb.2024.1452480 (PMC11629706; doi:10.3389/fcimb.2024.1452480)
Supplement: Supplementary file 1 [file DataSheet1.zip › mendelian test/ebi-a-GCST90027476.csv_forest.pdf]

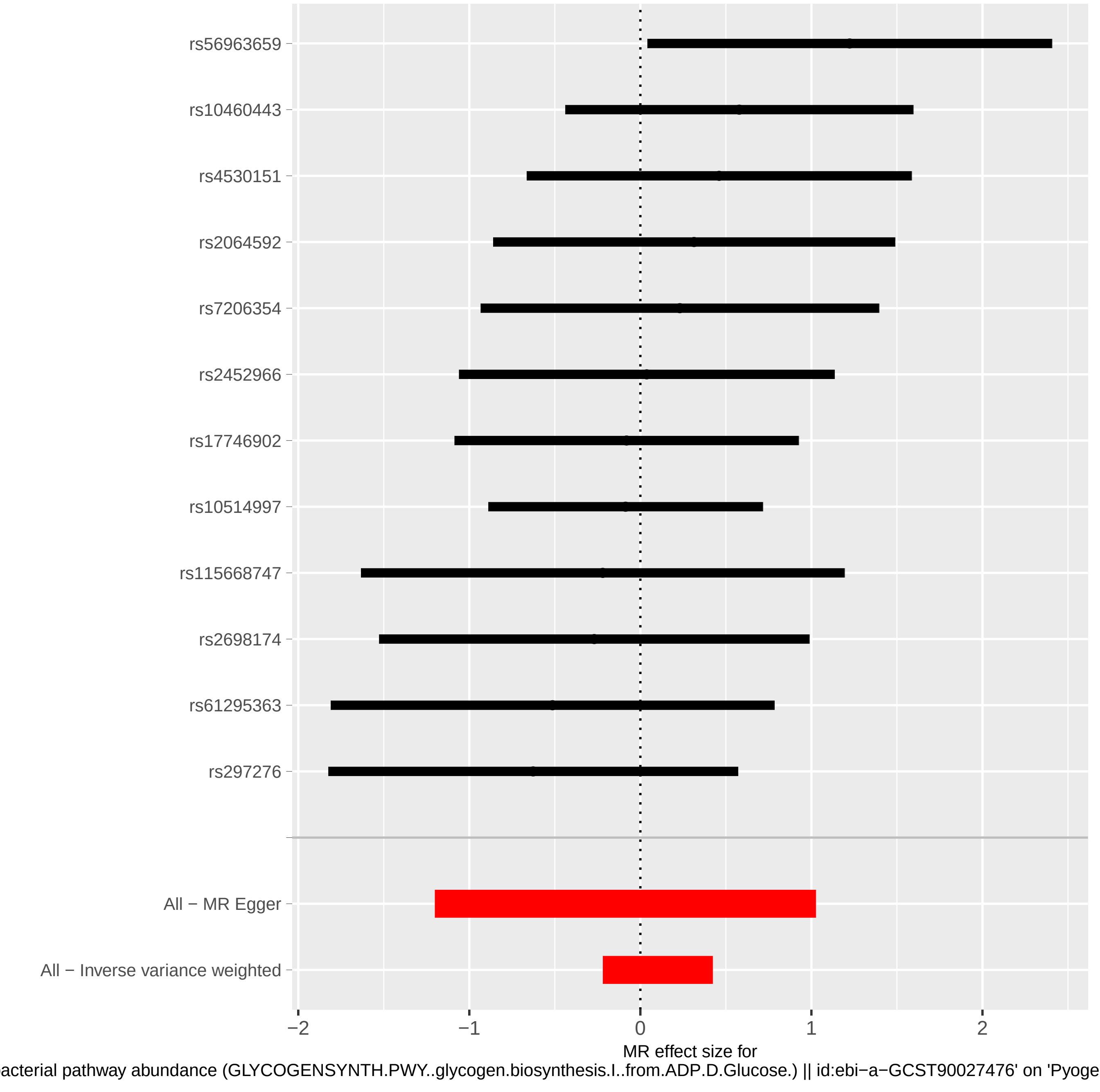
rs56963659
rs10460443
rs4530151
rs2064592
rs7206354
rs2452966
rs17746902
rs10514997
rs115668747
rs2698174
rs61295363
rs297276
All − MR Egger
All − Inverse variance weighted
−2
−1
0
1
2
MR effect size for
acterial pathway abundance (GLYCOGENSYNTH.PWY..glycogen.biosynthesis.I..from.ADP.D.Glucose.) || id:ebi−a−GCST90027476' on 'Pyoge
